# Bis(1,10-phenanthrolin-1-ium) tetra­chlorido­zincate monohydrate

**DOI:** 10.1107/S1600536814000208

**Published:** 2014-01-18

**Authors:** E. Govindan, Subramani Thirumurugan, Ayyakannu Sundaram Ganeshraja, Krishnamoorthy Anbalagan, A. SubbiahPandi

**Affiliations:** a Department of Physics, Presidency College (Autonomous), Chennai 600 005, India; b Department of Chemistry, Pondicherry University, Pondicherry 605 014, India

## Abstract

In the crystal structure of the title compound, (C_12_H_9_N_2_)_2_[ZnCl_4_]·H_2_O, the two independent 1,10-phenanthrolinium cations are bridged by the water mol­ecule and the tetrahedral tetrachloridozincate anion *via* N—H⋯O, O—H⋯Cl and N—H⋯Cl hydrogen bonds, forming chains along [100]. The chains are linked *via* C—H⋯Cl hydrogen bonds and a number of π–π inter­actions [centroid–centroid distances vary from 3.5594 (14) to 3.7057 (13) Å], forming a three-dimensional network. In each 1,10-phenanthrolinium cation, there is a short N—H⋯N inter­action.

## Related literature   

For an example of the crystal structure of a hybrid compound combining an organic cation and the tetrachloridozincate anion, see: Dong & Liu (2012 ▶). For details of the Cambridge Structural Database, see: Allen (2002 ▶).
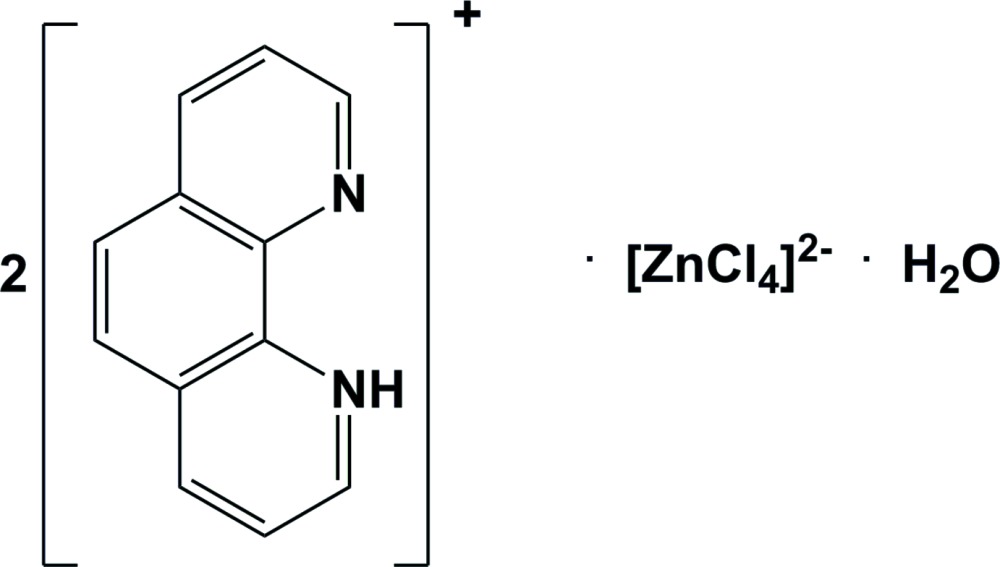


## Experimental   

### 

#### Crystal data  

(C_12_H_9_N_2_)_2_[ZnCl_4_]·H_2_O
*M* *_r_* = 587.61
Monoclinic, 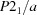

*a* = 14.6046 (5) Å
*b* = 10.8008 (3) Å
*c* = 16.3151 (6) Å
β = 107.390 (4)°
*V* = 2455.93 (14) Å^3^
*Z* = 4
Mo *K*α radiation
μ = 1.46 mm^−1^
*T* = 293 K
0.21 × 0.18 × 0.15 mm

#### Data collection  

Oxford Diffraction Xcalibur diffractometer with Eos detector
Absorption correction: multi-scan (*CrysAlis PRO*; Oxford Diffraction, 2009 ▶) *T*_min_ = 0.743, *T*_max_ = 0.803
10373 measured reflections
4293 independent reflections
3414 reflections with *I* > 2σ(*I*)
*R*_int_ = 0.028

#### Refinement  

*R*[*F* ^2^ > 2σ(*F* ^2^)] = 0.031
*wR*(*F* ^2^) = 0.068
*S* = 1.05
4293 reflections
324 parameters
H atoms treated by a mixture of independent and constrained refinement
Δρ_max_ = 0.38 e Å^−3^
Δρ_min_ = −0.29 e Å^−3^

### 

Data collection: *CrysAlis CCD* (Oxford Diffraction, 2009 ▶); cell refinement: *CrysAlis CCD*; data reduction: *CrysAlis RED* (Oxford Diffraction, 2009 ▶); program(s) used to solve structure: *SHELXS97* (Sheldrick, 2008 ▶); program(s) used to refine structure: *SHELXL97* (Sheldrick, 2008 ▶); molecular graphics: *ORTEP-3 for Windows* (Farrugia, 2012 ▶); software used to prepare material for publication: *SHELXL97* (Sheldrick, 2008 ▶) and *PLATON* (Spek, 2009 ▶).

## Supplementary Material

Crystal structure: contains datablock(s) global, I. DOI: 10.1107/S1600536814000208/su2681sup1.cif

Structure factors: contains datablock(s) I. DOI: 10.1107/S1600536814000208/su2681Isup2.hkl

CCDC reference:

Additional supporting information:  crystallographic information; 3D view; checkCIF report

## Figures and Tables

**Table 1:** Hydrogen-bond geometry (Å, °)

*D*—H⋯*A*	*D*—H	H⋯*A*	*D*⋯*A*	*D*—H⋯*A*
N1—H1⋯O1^i^	0.74 (3)	2.01 (3)	2.711 (4)	158 (2)
O1—H1*A*⋯Cl1	0.80 (4)	2.44 (4)	3.231 (3)	172 (3)
O1—H1*B*⋯Cl2^ii^	0.73 (3)	2.82 (4)	3.317 (3)	128 (4)
N15—H15⋯Cl3	0.83 (3)	2.50 (2)	3.225 (2)	146 (2)
C3—H3⋯Cl2^iii^	0.93	2.80	3.728 (3)	172
C24—H24⋯Cl2^iv^	0.93	2.74	3.629 (3)	160
N1—H1⋯N12	0.74 (3)	2.42 (2)	2.737 (3)	107 (2)
N15—H15⋯N26	0.83 (3)	2.41 (2)	2.731 (3)	104 (2)

Symmetry codes: (i) 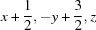; (ii) 
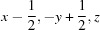
; (iii) 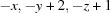; (iv) 
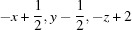
.

**Fig. 1.**
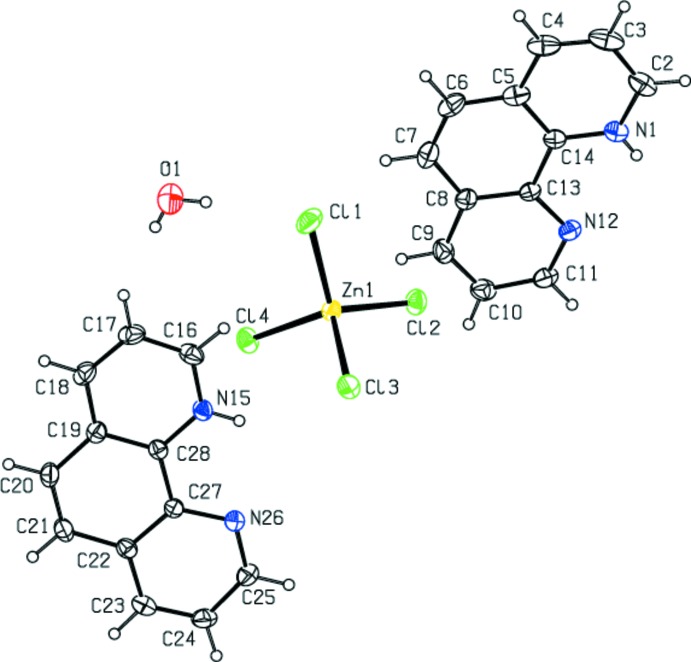
The molecular structure of the title compound, with the atom labelling. Displacement ellipsoids are drawn at 30% probability level.

**Fig. 2.**
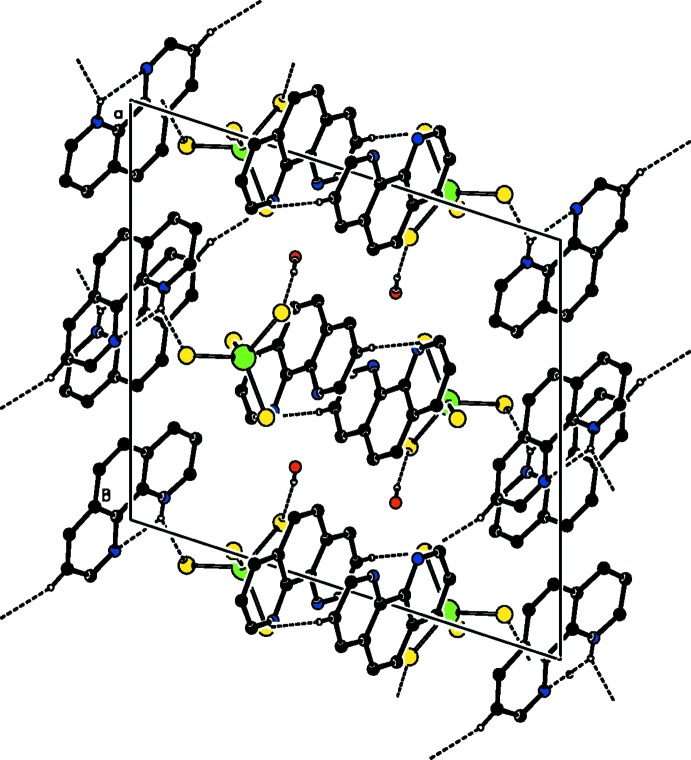
A view along the b-axis of the crystal packing of the title compound. Hydrogen bonds are shown as dashed lines (see Table 1 for details).

## supplementary crystallographic information

### 1. Comment

As part of an ongoing investigation of the structures of and non-covalent interactions present in self-assembling organic and inorganic hybrid materials prepared by the combination of an organic cation and the tetrachloridozincate anion we synthesized the title compound. There are only a small number of structures of materials containing bis(1,10-phenanthrolinium) cations and perhalometallate anions in the Cambridge Structural Database (CSD; V5.35, last update Nov. 2013; Allen, 2002), and none of them involve the tetrachloridozincate anion.

The molecule structure of the title compound is shown in Fig. 1. The asymmetric unit contains one inorganic tetrachloridozincate anion and two 1,10-phenanthrolinium organic cations. The compound crystallized as a monohydrate. The tetrachlorozincate anion has a perfect tetrahedral coordination environment. The bond lengths Zn—Cl [2.556 (7) - 2.3085 (7) Å] and C—N [1.320 (3) - 1.362 (3) Å] are comparable with the values reported for Bis(10-methoxybenzo[*h*]quinolinium) tetrachloridozinc [Dong & Liu, 2012]. The sum of the bond angles around atoms N1 and N15 (360°) in the 1,10-phenanthrolinium cations indicates *sp*^2^ hybridization states. The two 1,10-phenanthrolinium ring systems (N1/N12/C2-C11/C13/C14) and (N15/N26/C16-C25/C27/C28) are planar with r.m.s values of 0.029 (3) and 0.022 (2) Å, respectively. In each 1,10-phenanthrolinium cation there is a short N-H···N interaction (Table 1).

In the crystal, the two independent 1,10-phenanthrolinium cations are bridged by the water molecule and the tetrachloridozinc anion via N-H···O, O-H···Cl and N-H···Cl hydrogen bonds (Table 1 and Fig. 2) forming chains along [100]. The chains are linked via C-H···Cl hydrogen bonds (Table 1) and a number of π-π interactions forming a three-dimensional network.

The centroid-centroid distances are 3.5594 (14) Å for Cg1···Cg2^i^ [Cg1 and Cg2 and the centroids of rings N1/C2-C5/C14 and N12/C8-C11/C13, respectively; symmetry code: (i) = -x, -y+2, -z+1 ], 3.6501 (15) Å for Cg1···Cg3^i^ [Cg3 is the centroid of ring C5-C8/C13/C14] and 3.7057 (13) Å for Cg8···Cg9^ii^ [Cg8 and Cg9 are the centroids of rings N26/C22-C25/C27 and C19-C22/C27/C28, respectively; symmetry code: (ii) -x, -y, -z+2].

### 2. Experimental

Zinc chloride (136 mg, 1 mmol) was dissolved in 10 mL of water. To this 1,10-phenanthroline (396 mg, 2 mmol) in 20 ml of an EtOH/HCl mixture (1:9 *v*/*v*) was added drop wise. The mixture was heated to 323 K for 2–3 hrs and then allowed to stand. On slow evaporation colourless crystals separated out. They were filtered off and recrystallized using acidified water.

### 3. Refinement

The NH and water H atoms were located in a difference Fourier map and freely refined. The C bound H atoms were positioned geometrically and allowed to ride on their parent atoms: C–H = 0.93 Å with *U*_iso_(H) = 1.2*U*_eq_(C).

### Crystal data

**Table:**

(C_12_H_9_N_2_)_2_[ZnCl_4_]·H_2_O	*F*(000) = 1192
*M**_r_* = 587.61	*D*_x_ = 1.589 Mg m^−^^3^
Monoclinic, *P*2_1_/*a*	Mo *K*α radiation, λ = 0.71073 Å
Hall symbol: -P 2yab	Cell parameters from 3414 reflections
*a* = 14.6046 (5) Å	θ = 3.8–25.0°
*b* = 10.8008 (3) Å	µ = 1.46 mm^−^^1^
*c* = 16.3151 (6) Å	*T* = 293 K
β = 107.390 (4)°	Block, colourless
*V* = 2455.93 (14) Å^3^	0.21 × 0.18 × 0.15 mm
*Z* = 4	

### Data collection

**Table:**

Oxford Diffraction Xcalibur diffractometer with Eos detector	4293 independent reflections
Radiation source: fine-focus sealed tube	3414 reflections with *I* > 2σ(*I*)
Graphite monochromator	*R*_int_ = 0.028
ω and φ scans	θ_max_ = 25.0°, θ_min_ = 3.8°
Absorption correction: multi-scan (*CrysAlis PRO*; Oxford Diffraction, 2009)	*h* = −17→17
*T*_min_ = 0.743, *T*_max_ = 0.803	*k* = −11→12
10373 measured reflections	*l* = −19→19

### Refinement

**Table:**

Refinement on *F*^2^	Secondary atom site location: difference Fourier map
Least-squares matrix: full	Hydrogen site location: inferred from neighbouring sites
*R*[*F*^2^ > 2σ(*F*^2^)] = 0.031	H atoms treated by a mixture of independent and constrained refinement
*wR*(*F*^2^) = 0.068	*w* = 1/[σ^2^(*F*_o_^2^) + (0.0278*P*)^2^ + 0.2557*P*] where *P* = (*F*_o_^2^ + 2*F*_c_^2^)/3
*S* = 1.05	(Δ/σ)_max_ < 0.001
4293 reflections	Δρ_max_ = 0.38 e Å^−^^3^
324 parameters	Δρ_min_ = −0.29 e Å^−^^3^
0 restraints	Extinction correction: *SHELXL97* (Sheldrick, 2008), Fc^*^=kFc[1+0.001xFc^2^λ^3^/sin(2θ)]^-1/4^
Primary atom site location: structure-invariant direct methods	Extinction coefficient: 0.0165 (5)

### Special details

**Table:**

Geometry. Bond distances, angles etc. have been calculated using the rounded fractional coordinates. All su's are estimated from the variances of the (full) variance-covariance matrix. The cell esds are taken into account in the estimation of distances, angles and torsion angles
Refinement. Refinement of *F*^2^ against ALL reflections. The weighted *R*-factor *wR* and goodness of fit *S* are based on *F*^2^, conventional *R*-factors *R* are based on *F*, with *F* set to zero for negative *F*^2^. The threshold expression of *F*^2^ > σ(*F*^2^) is used only for calculating *R*-factors(gt) *etc*. and is not relevant to the choice of reflections for refinement. *R*-factors based on *F*^2^ are statistically about twice as large as those based on *F*, and *R*- factors based on ALL data will be even larger.

### Fractional atomic coordinates and isotropic or equivalent isotropic displacement parameters (Å^2^)

**Table:**

	*x*	*y*	*z*	*U*_iso_*/*U*_eq_	
N1	0.05391 (18)	1.17583 (19)	0.56437 (14)	0.0382 (8)	
N12	0.13002 (14)	0.97933 (17)	0.66837 (13)	0.0364 (7)	
C2	0.0227 (2)	1.2729 (2)	0.51338 (17)	0.0498 (10)	
C3	−0.0746 (2)	1.2890 (3)	0.47489 (18)	0.0574 (10)	
C4	−0.1379 (2)	1.2056 (3)	0.49016 (18)	0.0533 (11)	
C5	−0.10528 (18)	1.1038 (2)	0.54406 (16)	0.0401 (9)	
C6	−0.16693 (19)	1.0148 (3)	0.56456 (18)	0.0497 (10)	
C7	−0.13202 (18)	0.9219 (3)	0.61895 (18)	0.0475 (10)	
C8	−0.03095 (17)	0.9048 (2)	0.65657 (15)	0.0354 (8)	
C9	0.0097 (2)	0.8067 (2)	0.71173 (17)	0.0437 (9)	
C10	0.1068 (2)	0.7959 (2)	0.74208 (18)	0.0469 (10)	
C11	0.16361 (19)	0.8837 (2)	0.71874 (16)	0.0427 (9)	
C13	0.03328 (16)	0.9889 (2)	0.63748 (14)	0.0293 (7)	
C14	−0.00593 (17)	1.0909 (2)	0.58142 (14)	0.0306 (8)	
N15	−0.07856 (15)	0.34171 (18)	0.92218 (13)	0.0339 (7)	
N26	0.08664 (14)	0.25794 (18)	1.03725 (13)	0.0378 (7)	
C16	−0.15449 (18)	0.3854 (2)	0.86201 (17)	0.0424 (9)	
C17	−0.24346 (18)	0.3314 (3)	0.84870 (17)	0.0460 (9)	
C18	−0.25201 (17)	0.2333 (2)	0.89907 (17)	0.0428 (9)	
C19	−0.17174 (16)	0.1862 (2)	0.96188 (15)	0.0339 (8)	
C20	−0.17495 (18)	0.0817 (2)	1.01531 (17)	0.0411 (9)	
C21	−0.09527 (18)	0.0398 (2)	1.07310 (16)	0.0407 (9)	
C22	−0.00318 (17)	0.0967 (2)	1.08369 (15)	0.0327 (8)	
C23	0.08261 (19)	0.0549 (2)	1.14257 (16)	0.0417 (9)	
C24	0.16602 (19)	0.1130 (2)	1.14723 (17)	0.0473 (9)	
C25	0.16456 (18)	0.2136 (3)	1.09347 (18)	0.0470 (9)	
C27	0.00304 (16)	0.1986 (2)	1.03291 (15)	0.0291 (7)	
C28	−0.08273 (16)	0.2437 (2)	0.97183 (15)	0.0287 (7)	
Zn1	0.02573 (2)	0.43561 (2)	0.73719 (2)	0.0324 (1)	
Cl1	−0.11018 (5)	0.52315 (6)	0.64898 (5)	0.0584 (3)	
Cl2	0.15128 (4)	0.45542 (6)	0.68453 (4)	0.0445 (2)	
Cl3	0.06504 (5)	0.53130 (5)	0.86969 (4)	0.0429 (2)	
Cl4	−0.00552 (5)	0.23479 (5)	0.76075 (4)	0.0433 (2)	
O1	−0.25358 (17)	0.2883 (3)	0.61679 (19)	0.0609 (9)	
H1	0.1064 (18)	1.169 (2)	0.5852 (17)	0.033 (8)*	
H2	0.06620	1.32970	0.50370	0.0600*	
H3	−0.09690	1.35620	0.43880	0.0690*	
H4	−0.20340	1.21660	0.46450	0.0640*	
H6	−0.23290	1.02140	0.53950	0.0600*	
H7	−0.17430	0.86710	0.63270	0.0570*	
H9	−0.02950	0.74930	0.72740	0.0530*	
H10	0.13470	0.73060	0.77800	0.0560*	
H11	0.22980	0.87460	0.74000	0.0510*	
H15	−0.0270 (18)	0.379 (2)	0.9296 (16)	0.040 (8)*	
H16	−0.14770	0.45270	0.82870	0.0510*	
H17	−0.29670	0.36120	0.80630	0.0550*	
H18	−0.31190	0.19750	0.89150	0.0510*	
H20	−0.23320	0.04250	1.00970	0.0490*	
H21	−0.09940	−0.02770	1.10720	0.0490*	
H23	0.08200	−0.01220	1.17810	0.0500*	
H24	0.22340	0.08620	1.18570	0.0570*	
H25	0.22260	0.25230	1.09750	0.0560*	
H1A	−0.217 (3)	0.344 (3)	0.620 (2)	0.083 (15)*	
H1B	−0.239 (3)	0.241 (3)	0.650 (2)	0.079 (16)*	

### Atomic displacement parameters (Å^2^)

**Table:**

	*U*^11^	*U*^22^	*U*^33^	*U*^12^	*U*^13^	*U*^23^
N1	0.0427 (15)	0.0374 (13)	0.0337 (13)	0.0058 (11)	0.0103 (11)	0.0005 (10)
N12	0.0342 (11)	0.0336 (11)	0.0368 (12)	0.0014 (9)	0.0034 (9)	−0.0013 (9)
C2	0.081 (2)	0.0349 (15)	0.0369 (17)	0.0056 (14)	0.0228 (15)	0.0018 (12)
C3	0.087 (2)	0.0461 (17)	0.0368 (17)	0.0339 (17)	0.0149 (16)	0.0047 (13)
C4	0.0546 (18)	0.066 (2)	0.0358 (17)	0.0293 (16)	0.0081 (14)	−0.0022 (14)
C5	0.0395 (15)	0.0489 (15)	0.0312 (15)	0.0145 (12)	0.0096 (12)	−0.0083 (12)
C6	0.0301 (14)	0.0680 (19)	0.0472 (18)	0.0052 (14)	0.0058 (13)	−0.0136 (15)
C7	0.0374 (15)	0.0549 (17)	0.0524 (18)	−0.0112 (13)	0.0168 (13)	−0.0128 (14)
C8	0.0396 (14)	0.0367 (13)	0.0299 (14)	−0.0049 (11)	0.0105 (11)	−0.0108 (11)
C9	0.0582 (18)	0.0342 (14)	0.0405 (16)	−0.0133 (13)	0.0173 (14)	−0.0057 (12)
C10	0.0600 (19)	0.0330 (14)	0.0402 (16)	0.0000 (13)	0.0036 (14)	0.0023 (12)
C11	0.0407 (15)	0.0394 (14)	0.0388 (16)	0.0024 (12)	−0.0019 (12)	−0.0012 (12)
C13	0.0325 (13)	0.0291 (12)	0.0248 (13)	0.0011 (10)	0.0062 (10)	−0.0079 (10)
C14	0.0365 (13)	0.0317 (13)	0.0241 (13)	0.0034 (11)	0.0099 (11)	−0.0065 (10)
N15	0.0305 (12)	0.0374 (12)	0.0362 (13)	0.0010 (10)	0.0138 (10)	0.0036 (10)
N26	0.0319 (11)	0.0453 (12)	0.0355 (13)	−0.0040 (9)	0.0090 (9)	0.0053 (10)
C16	0.0423 (15)	0.0481 (15)	0.0396 (16)	0.0136 (13)	0.0164 (12)	0.0134 (12)
C17	0.0326 (15)	0.0642 (18)	0.0382 (16)	0.0129 (13)	0.0059 (12)	0.0039 (14)
C18	0.0282 (13)	0.0558 (17)	0.0437 (17)	−0.0009 (12)	0.0098 (12)	−0.0058 (13)
C19	0.0320 (13)	0.0379 (13)	0.0340 (15)	−0.0021 (11)	0.0131 (11)	−0.0053 (11)
C20	0.0382 (15)	0.0454 (15)	0.0435 (16)	−0.0125 (12)	0.0180 (13)	−0.0040 (13)
C21	0.0482 (16)	0.0377 (14)	0.0404 (16)	−0.0082 (12)	0.0195 (13)	0.0033 (12)
C22	0.0389 (14)	0.0338 (13)	0.0277 (13)	0.0002 (11)	0.0137 (11)	−0.0008 (11)
C23	0.0520 (17)	0.0420 (15)	0.0316 (15)	0.0045 (13)	0.0133 (12)	0.0067 (12)
C24	0.0388 (15)	0.0594 (17)	0.0373 (16)	0.0056 (13)	0.0015 (12)	0.0101 (14)
C25	0.0305 (14)	0.0625 (18)	0.0444 (17)	−0.0057 (13)	0.0057 (12)	0.0052 (14)
C27	0.0302 (12)	0.0321 (13)	0.0264 (13)	−0.0010 (10)	0.0106 (10)	−0.0030 (10)
C28	0.0336 (13)	0.0285 (12)	0.0267 (13)	−0.0002 (10)	0.0132 (10)	−0.0025 (10)
Zn1	0.0315 (2)	0.0318 (2)	0.0336 (2)	−0.0033 (1)	0.0091 (1)	−0.0023 (1)
Cl1	0.0373 (4)	0.0486 (4)	0.0735 (5)	−0.0010 (3)	−0.0074 (3)	0.0052 (4)
Cl2	0.0409 (4)	0.0505 (4)	0.0470 (4)	−0.0073 (3)	0.0208 (3)	−0.0059 (3)
Cl3	0.0575 (4)	0.0369 (3)	0.0369 (4)	−0.0085 (3)	0.0182 (3)	−0.0086 (3)
Cl4	0.0575 (4)	0.0299 (3)	0.0460 (4)	−0.0072 (3)	0.0210 (3)	−0.0050 (3)
O1	0.0530 (14)	0.0493 (14)	0.083 (2)	0.0033 (13)	0.0242 (13)	0.0021 (14)

### Geometric parameters (Å, º)

**Table:**

Zn1—Cl4	2.2728 (6)	C2—H2	0.9300
Zn1—Cl1	2.2798 (8)	C3—H3	0.9300
Zn1—Cl2	2.2556 (7)	C4—H4	0.9300
Zn1—Cl3	2.3085 (7)	C6—H6	0.9300
O1—H1A	0.80 (4)	C7—H7	0.9300
O1—H1B	0.73 (3)	C9—H9	0.9300
N1—C2	1.331 (3)	C10—H10	0.9300
N1—C14	1.352 (3)	C11—H11	0.9300
N12—C13	1.355 (3)	C16—C17	1.381 (4)
N12—C11	1.320 (3)	C17—C18	1.369 (4)
N1—H1	0.74 (3)	C18—C19	1.402 (3)
N15—C28	1.345 (3)	C19—C20	1.435 (3)
N15—C16	1.329 (3)	C19—C28	1.406 (3)
N26—C25	1.320 (4)	C20—C21	1.339 (4)
N26—C27	1.362 (3)	C21—C22	1.441 (4)
N15—H15	0.83 (3)	C22—C27	1.397 (3)
C2—C3	1.382 (4)	C22—C23	1.406 (4)
C3—C4	1.366 (4)	C23—C24	1.352 (4)
C4—C5	1.400 (4)	C24—C25	1.393 (4)
C5—C6	1.424 (4)	C27—C28	1.433 (3)
C5—C14	1.403 (4)	C16—H16	0.9300
C6—C7	1.336 (4)	C17—H17	0.9300
C7—C8	1.431 (4)	C18—H18	0.9300
C8—C13	1.406 (3)	C20—H20	0.9300
C8—C9	1.402 (3)	C21—H21	0.9300
C9—C10	1.360 (4)	C23—H23	0.9300
C10—C11	1.386 (4)	C24—H24	0.9300
C13—C14	1.437 (3)	C25—H25	0.9300
			
Cl3—Zn1—Cl4	105.98 (2)	C8—C7—H7	119.00
Cl1—Zn1—Cl4	108.76 (3)	C8—C9—H9	120.00
Cl1—Zn1—Cl2	111.95 (3)	C10—C9—H9	120.00
Cl1—Zn1—Cl3	109.35 (3)	C9—C10—H10	120.00
Cl2—Zn1—Cl3	108.14 (3)	C11—C10—H10	120.00
Cl2—Zn1—Cl4	112.47 (3)	N12—C11—H11	118.00
H1A—O1—H1B	116 (4)	C10—C11—H11	118.00
C2—N1—C14	122.8 (3)	N15—C16—C17	120.3 (2)
C11—N12—C13	116.3 (2)	C16—C17—C18	118.9 (2)
C14—N1—H1	118.7 (18)	C17—C18—C19	120.9 (2)
C2—N1—H1	118.5 (18)	C18—C19—C28	117.8 (2)
C16—N15—C28	123.1 (2)	C18—C19—C20	123.9 (2)
C25—N26—C27	116.0 (2)	C20—C19—C28	118.3 (2)
C28—N15—H15	119.8 (16)	C19—C20—C21	121.0 (2)
C16—N15—H15	117.1 (16)	C20—C21—C22	121.6 (2)
N1—C2—C3	119.8 (3)	C21—C22—C27	119.3 (2)
C2—C3—C4	119.6 (3)	C23—C22—C27	117.1 (2)
C3—C4—C5	120.7 (3)	C21—C22—C23	123.6 (2)
C4—C5—C14	117.7 (2)	C22—C23—C24	119.6 (2)
C4—C5—C6	123.9 (3)	C23—C24—C25	118.9 (2)
C6—C5—C14	118.4 (2)	N26—C25—C24	124.7 (3)
C5—C6—C7	121.4 (3)	C22—C27—C28	118.8 (2)
C6—C7—C8	121.5 (3)	N26—C27—C28	117.4 (2)
C9—C8—C13	116.6 (2)	N26—C27—C22	123.8 (2)
C7—C8—C13	119.5 (2)	C19—C28—C27	121.1 (2)
C7—C8—C9	123.9 (2)	N15—C28—C19	119.0 (2)
C8—C9—C10	119.6 (2)	N15—C28—C27	119.9 (2)
C9—C10—C11	119.1 (2)	N15—C16—H16	120.00
N12—C11—C10	124.4 (3)	C17—C16—H16	120.00
C8—C13—C14	118.1 (2)	C18—C17—H17	121.00
N12—C13—C14	117.9 (2)	C16—C17—H17	121.00
N12—C13—C8	124.0 (2)	C17—C18—H18	120.00
N1—C14—C5	119.4 (2)	C19—C18—H18	120.00
N1—C14—C13	119.5 (2)	C21—C20—H20	120.00
C5—C14—C13	121.1 (2)	C19—C20—H20	120.00
N1—C2—H2	120.00	C20—C21—H21	119.00
C3—C2—H2	120.00	C22—C21—H21	119.00
C4—C3—H3	120.00	C22—C23—H23	120.00
C2—C3—H3	120.00	C24—C23—H23	120.00
C3—C4—H4	120.00	C23—C24—H24	121.00
C5—C4—H4	120.00	C25—C24—H24	121.00
C5—C6—H6	119.00	N26—C25—H25	118.00
C7—C6—H6	119.00	C24—C25—H25	118.00
C6—C7—H7	119.00		
			
C14—N1—C2—C3	−0.6 (4)	C8—C9—C10—C11	1.0 (4)
C2—N1—C14—C13	−179.7 (2)	C9—C10—C11—N12	0.2 (4)
C2—N1—C14—C5	0.3 (4)	C8—C13—C14—C5	−2.4 (3)
C11—N12—C13—C14	−179.1 (2)	N12—C13—C14—C5	177.2 (2)
C11—N12—C13—C8	0.5 (3)	C8—C13—C14—N1	177.6 (2)
C13—N12—C11—C10	−0.9 (4)	N12—C13—C14—N1	−2.7 (3)
C16—N15—C28—C27	177.3 (2)	N15—C16—C17—C18	0.7 (4)
C28—N15—C16—C17	1.0 (4)	C16—C17—C18—C19	−1.4 (4)
C16—N15—C28—C19	−1.9 (3)	C17—C18—C19—C28	0.6 (4)
C25—N26—C27—C28	−179.4 (2)	C17—C18—C19—C20	−178.3 (2)
C25—N26—C27—C22	0.0 (3)	C20—C19—C28—C27	0.8 (3)
C27—N26—C25—C24	−0.3 (4)	C18—C19—C20—C21	178.7 (2)
N1—C2—C3—C4	0.6 (4)	C28—C19—C20—C21	−0.1 (4)
C2—C3—C4—C5	−0.3 (4)	C18—C19—C28—C27	−178.2 (2)
C3—C4—C5—C14	0.0 (4)	C20—C19—C28—N15	180.0 (2)
C3—C4—C5—C6	178.5 (3)	C18—C19—C28—N15	1.1 (3)
C4—C5—C14—N1	0.0 (4)	C19—C20—C21—C22	−0.5 (4)
C4—C5—C6—C7	−177.5 (3)	C20—C21—C22—C23	−179.0 (2)
C14—C5—C6—C7	1.0 (4)	C20—C21—C22—C27	0.5 (4)
C4—C5—C14—C13	−180.0 (2)	C23—C22—C27—N26	0.3 (3)
C6—C5—C14—C13	1.5 (3)	C21—C22—C27—N26	−179.2 (2)
C6—C5—C14—N1	−178.6 (2)	C21—C22—C27—C28	0.2 (3)
C5—C6—C7—C8	−2.4 (4)	C23—C22—C27—C28	179.7 (2)
C6—C7—C8—C9	−177.8 (3)	C21—C22—C23—C24	179.1 (2)
C6—C7—C8—C13	1.4 (4)	C27—C22—C23—C24	−0.5 (3)
C7—C8—C9—C10	177.8 (3)	C22—C23—C24—C25	0.3 (4)
C9—C8—C13—N12	0.6 (3)	C23—C24—C25—N26	0.2 (4)
C7—C8—C13—N12	−178.6 (2)	N26—C27—C28—N15	−0.6 (3)
C9—C8—C13—C14	−179.7 (2)	C22—C27—C28—C19	−0.8 (3)
C7—C8—C13—C14	1.0 (3)	N26—C27—C28—C19	178.6 (2)
C13—C8—C9—C10	−1.4 (4)	C22—C27—C28—N15	−180.0 (2)

### Hydrogen-bond geometry (Å, º)

**Table:**

*D*—H···*A*	*D*—H	H···*A*	*D*···*A*	*D*—H···*A*
N1—H1···O1^i^	0.74 (3)	2.01 (3)	2.711 (4)	158 (2)
O1—H1*A*···Cl1	0.80 (4)	2.44 (4)	3.231 (3)	172 (3)
O1—H1*B*···Cl2^ii^	0.73 (3)	2.82 (4)	3.317 (3)	128 (4)
N15—H15···Cl3	0.83 (3)	2.50 (2)	3.225 (2)	146 (2)
C3—H3···Cl2^iii^	0.93	2.80	3.728 (3)	172
C24—H24···Cl2^iv^	0.93	2.74	3.629 (3)	160
N1—H1···N12	0.74 (3)	2.42 (2)	2.737 (3)	107 (2)
N15—H15···N26	0.83 (3)	2.41 (2)	2.731 (3)	104 (2)

Symmetry codes: (i) *x*+1/2, −*y*+3/2, *z*; (ii) *x*−1/2, −*y*+1/2, *z*; (iii) −*x*, −*y*+2, −*z*+1; (iv) −*x*+1/2, *y*−1/2, −*z*+2.
